# Undergraduate teaching of urology: *Quo vadis?*

**DOI:** 10.1097/j.pbj.0000000000000135

**Published:** 2021-06-14

**Authors:** Diogo Pereira, Raquel Catarino, Vasco Rodrigues, Gabriel Costa, João Silva, Frederico Carmo-Reis, Carlos Martins-Silva

**Affiliations:** a Urology Department, Unidade Local de Saúde de Matosinhos, Hospital Pedro Hispano, Matosinhos; b Urology Department, Centro Hospitalar Universitário de São João; c Faculty of Medicine of University of Porto, Porto, Portugal.

**Keywords:** questionnaires, undergraduate medical education, urology

## Abstract

**Background:**

The undergraduate teaching of urology is not uniform in the various European medical schools and even absent in some of them, despite the widespread adoption of the Bologna process, which advocates a standardization and harmonization of medical education. Our aim was to evaluate the perception of junior doctors about the undergraduate teaching of Urology and the exposure to the specialty of Urology in undergraduate education in Portuguese medical schools.

**Methods:**

A questionnaire was emailed to all physicians who first enrolled in the Board of Portuguese Doctors in 2017 and 2018. The questionnaire consisted of several questions about specialty exposure, pathology, and basic urological procedures. A database for statistical analysis was created.

**Results:**

One hundred and eighty-six answers were considered valid. Although almost all participant physicians attribute considerable importance to Urology specialty, most find their exposure to urological pathology and basic urological procedures to be inappropriate in medical school. Urinary lithiasis and lower urinary tract symptoms are the subjects on which doctors feel most prepared after graduating. Interestingly, 63.4% of doctors consider that the education they had in college was preponderant in choosing their specialty.

**Conclusions:**

The teaching of Urology in Portuguese Medical Schools is considered by junior doctors as inadequate, not reflecting the importance of this specialty in the clinical practice. These results are like those found in other countries. A reflection and consequent change of the teaching paradigm is necessary, namely at the practical teaching level.

## Abstract 2

Supplemental Digital Content is available in the text

## Introduction

In 1999, many European Ministers of Education signed the Pact of Convergence of Bologna, whose main objectives were to harmonize and standardize medicine teaching in all European Medical Schools.^1^

However, the undergraduate teaching of urology is not uniform in the variety of Medical Schools and paradoxically is even absent in some of them. For example in North America, different studies have shown a decline in urologic education among medical students during the last years.^2–4^ As consequence, their ability in dealing with common urological diseases and basic procedures have been shown to be inconstant and inadequate in many cases. Concerning this issue, studies from Canada and United of States have demonstrated the need of urgent changes in teaching of basic urological notions.^5,6^

Urological diseases are highly prevalent in the real-life clinical practice of general practitioners, and their incidence tends to increase with population aging. Therefore, undergraduate teaching of urology basic knowledge seems to be essential, enabling future doctors for patient care in the general clinical practice.

The purpose of this paper was to assess medical perception about undergraduate teaching of urology in Portuguese Medical Schools and young doctors’ experience and comfort in performing basic urological procedures and dealing with urological diseases.

## Material and methods

Junior Portuguese doctors that had registered in the Portuguese Medical Association in 2017 and 2018 were invited to participate in the study via email. A questionnaire was sent to them with a range of questions regarding their contact with the specialty, urological pathology and basic urological procedures during Medical School (see Appendix). The questionnaire was developed using Google Forms (https://docs.google.com/forms/).

All questionnaire data were recorded electronically and anonymously. Categorical variables presented as frequencies and percentages were analyzed using the chi-square test or Fisher's exact test, as appropriate. All reported *P* values were 2-sided, with a *P* value of <.05 indicating statistical significance. Statistical analysis was performed with IBM Statistical Package for Social Science^®^ (IBM SPSS version 25.0).

## Results

We obtained responses from 189 medical doctors. Three of them were excluded because they graduated in foreign schools, so a total of 186 valid responses were considered from doctors of all Portuguese Medical Schools. Faculdade de Medicina da Universidade de Lisboa, Faculdade de Medicina da Universidade do Porto and Nova Medical School—Faculdade de Ciências Médicas were the schools from which we obtained more participations (Table 1).

**Table 1:** Characteristics of applicants who participated in the study and their contact with urology

	n (%)
Total of residents	186
*Departamento de Ciências Biomédicas e Medicina da Universidade do Algarve*	5 (2.7)
*Escola Medicina Universidade do Minho*	11 (5.9)
*Faculdade de Ciências da Saúde da Universidade da Beira Interior*	13 (7.0)
*Faculdade de Medicina da Universidade de Coimbra*	28 (15.1)
*Faculdade de Medicina da Universidade de Lisboa*	43 (23.1)
*Faculdade de Medicina da Universidade do Porto*	31 (16.7)
*Instituto de Ciências Biomédicas Abel Salazar*	24 (12.9)
Nova Medical School	31 (16.7)
Year of graduation	
2017	85 (45.7)
2018	101 (54.3)
Importance of urology	
Little important	3 (1.6)
Important	110 (59.1)
Very important	73 (39.2)
Exposure to urological pathology and basic procedures	
Adequate	68 (36.6)
Inadequate	118 (63.4)
Familiar with	
Scrotal pain	85 (45.9)
Epididymitis	28 (15.1)
Prostatitis	80 (43.2)
Renal colic	182 (98.4)
Urinary incontinence	112 (60.5)
Better-prepared urological condition	
Urolithiasis	93 (50)
Lower urinary tract symptoms	76 (40.9)
Urologic oncology	2 (1.1)
Erectile dysfunction	2 (1.1)
Urinary incontinence	12 (6.5)
Kidney transplantation	1 (0.5)
Type of classes	
Theoretical	186 (100)
Practical	123 (66.8)
Case clinical discussion	96 (54.5)
Technologies	
Online learning material	93 (50.5)
Interactive cases	56 (30.4)
Uroradiological cases	70 (38.5)
Videos of technical procedures	53 (29.3)
Urology clerkship	21 (11.3)
Doctors who considered being a urologist	79 (42.5)
Influence of teaching at medical school in the choice of specialty	
Yes	116 (62.4)
No	70 (37.6)

Almost all doctors considered Urology a clinically important or very important specialty (59.3% and 39.2%, respectively). However, 63.4% thought they had an inadequate exposure to urological pathology and basic procedures as students in the medical school.

Urolithiasis (50.3%) and lower urinary tract symptoms (40.7%) are the topics that doctors felt better prepared to deal with. Important clinical scenarios in which most of residents did not feel familiar with include epididymitis, prostatitis and scrotal pain.

**Figure 1:**
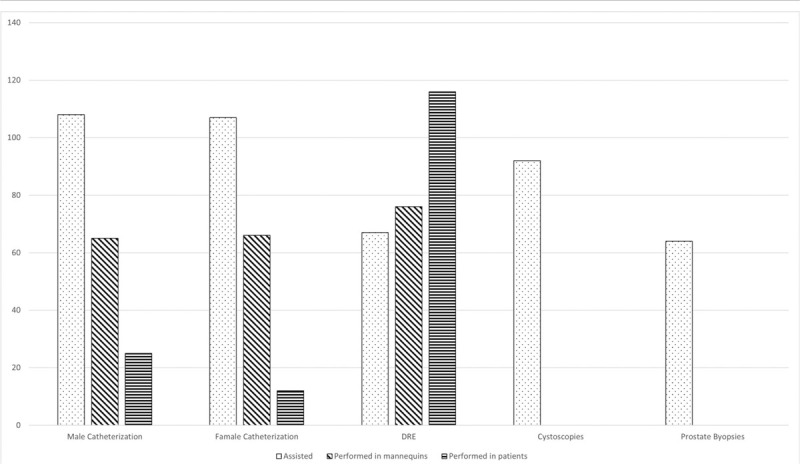
Contact with urological procedures. DRE = digital rectal exam. The parameters are expressed as n-absolute number.

Figure 1 demonstrates the urological procedures with which residents have contacted with. Hands-on teaching approaches have increased in Portuguese Medical Schools in the past years: around 34% of doctors have done transurethral catheterization while 40.2% have done a rectal digital exam in mannequins.

Sixty-two (33.3%) residents did not have practical classes addressing urological issues. Our results demonstrate an association between attending practical classes and the perception of an adequate exposure to urological pathology and basic procedures (*P* < .001) (Table 2). Furthermore, we found a statistically significant association between perception of an adequate exposure and performing practical procedures, namely male mannequin catheterization (*P* = .012), female mannequin catheterization (*P* = .017), in vivo male catheterization (*P* < .001) and in vivo digital rectal exam (*P* = .013).

**Table 2:** Association between practice and the perception of exposure and the consideration of being urologist in the future

	Perception of exposure			Urology as future specialty		
		
	Adequate	Inadequate	*P* value	Considered	Not Considered	*P* value
Practical classes						
Yes	64	60	<.001	59	65	.046
No	4	58		20	42	
In vivo male catheterization						
Yes	21	4	<.001	17	8	.006
No	47	114		62	99	
Mannequin male catheterization						
Yes	32	33	.009	30	35	.457
No	36	85		49	72	
In vivo female catheterization						
Yes	7	5	.127	9	3	.018
No	61	113		70	104	
Mannequin female catheterization						
Yes	32	34	.012	29	37	.764
No	36	84		50	70	
In vivo digital rectal examination						
Yes	49	64	.017	55	58	.033
No	19	54		24	49	
Mannequin digital rectal examination						
Yes	30	46	.493	32	44	.933
No	38	72		47	63	
Cystoscopy						
Yes	45	47	.001	44	48	.144
No	23	71		35	59	
Urodynamics						
Yes	37	34	.001	38	33	.017
No	31	84		41	74	
Prostate biopsies						
Yes	33	31	.002	31	33	.233
No	35	87		48	74	

The parameters are expressed as n-absolute number.

Eighty-two (42.5%) residents considered choosing urology as their future specialty. Our results demonstrate that students who have had practical classes have a 4-fold higher likelihood of considering choosing urology as their specialty in the future. (OR = 3972, *P* = .046)

Interestingly, 62.4% of doctors considered that the undergraduate teaching they had was preponderant in choosing their specialty.

## Discussion

This study provides an overview of the undergraduate teaching of Urology in Portugal. We chose to query junior doctors rather than medical students, as they have recently completed their graduation and already have some degree of clinical experience. This questionnaire addressing residents’ opinion instead of testing their knowledge, allow us to get some conclusions.

Urology is well regarded by residents, with almost all considering it as an important or very important discipline and 43% considered the possibility of being a urologist. Unlike in USA, where the number of medical schools requiring urology classes is decreasing since 1950 until 5%, in all Portuguese Medical Schools urology classes are mandatory. In Portugal, they are usually introduced in the 5th year and the duration of urology exposure is between 30 to 60 hours, most of them included in an individual subject.

Nevertheless, most of the junior Portuguese doctors (63.4%) considered their exposure to urological pathology and procedures as inadequate. These results are worse than those from a study in Canada, where 44% of medical students considered their urological education insufficient.^5^ The exponential growing number of medical students and therefore larger classes, with a decrease of the practical component, can help to explain at least partially this complaint. Just as an example, more and more students complete their medical school without ever having performed a rectal digital exam or placing a transurethral bladder catheter.

Improving the way of teaching can also be part of the solution. The American Urological Association has developed online learning material such as podcasts, slide presentations and interactive case-scenarios. However, this is not equivalent to effective acquisition of knowledge. Kerfoot and Turek showed that traditional reading materials and face-to-face teaching were most helpful.^7^ In our sample, we saw that boot camps with mannequins can also work as a good teaching tool. Perhaps a combination of online resources, face-to-face interactions and boot camps with mannequins is the best option to improve urology teaching.

The low levels of comfort dealing with common urological diseases and urological basic procedures are concerning, especially because these pathologies are highly prevalent with an important social and economic burden and are likely to increase with population aging. Azer et al identified frequent urological diseases in the general clinical practice in which Australian residents were not able to deal with, like prostatitis, epididymitis and scrotal pain.^8^ These results are concordant to our results, in which most of the residents did not feel comfortable dealing with these pathologies.

Several factors should be considered when interpreting the survey results and we cannot exclude a response bias in our results, which is the main limitation. Our study is one of the first studies in Europe assessing Urologic Medical Education with questionnaires, and the first in Portugal. Being a Standardized undergraduate teaching in Europe an aim, further studies in this field are needed with valid questionnaires.

## Conclusion

A considerable proportion of Portuguese medical students had no urological practical education, reflecting an inadequate exposure in the medical school to urological diseases. It is therefore necessary to debate and consequently change the teaching paradigm, especially at the practical level.

## Assistance

None

## Conflicts of interest

None.

## Supplementary Material

Supplemental Digital Content
