# Supplementary material for: Undergraduate teaching of urology: Quo vadis?
Source: Porto Biomed J. 2021 Jun 14;6(3):e135. doi: 10.1097/j.pbj.0000000000000135 (PMC8202532; doi:10.1097/j.pbj.0000000000000135)
Supplement: Supplemental Digital Content [file pj9-6-e135-s001.doc]

**Appendix:** Urology Undergraduate teaching Questionnaire

1 – Which Medical School did you attend?

2- In which year did you graduate?

3- How do you categorize Urology as a specialty?

1. Medical Specialty
2. Surgical Specialty
3. Medical-Surgical Specialty

4- How important is Urology as a specialty?

1. Little important
2. Important
3. Very important

5- Indicate which urological procedures have you had the opportunity to assist / perform during Medical School:

1. *In vivo* male catheterization
2. Mannequin male catheterization
3. *In vivo* female catheterization
4. Mannequin female catheterization
5. *In vivo* digital Rectal Examination
6. Mannequin digital Rectal Examination
7. Suprapubic catheterization
8. Cystoscopy
9. Urological surgeries
10. Urodynamics
11. Prostate biopsies

6- What is your perception about your exposure to urological pathology and basic procedures?

1. Adequate
2. Inadequate

7- Regarding your exposure to urological pathology and basic urological procedures, do you think it has influenced your specialty choice?

1. Yes
2. No

8- Do you feel familiar with

1. Scrotal Pain? Yes / No
2. Epididymitis? Yes / No
3. Prostatitis? Yes / No
4. Renal Colic? Yes / No
5. Urinary Incontinence? Yes / No

9- Do you think you have been prepared for the most common urological clinical conditions?

1. Yes
2. No

10- Have you contacted with:

1. Online learning material? Yes / No
2. Interactive cases? Yes / No
3. Uroradiological cases? Yes / No
4. Videos of technical procedures? Yes / No

11- With which urological condition do you feel most prepared dealing with based on the knowledge acquired in Medical School?

1. Urolithiasis
2. Lower Urinary Tract Symptoms
3. Urologic Oncology
4. Erectile Dysfunction
5. Kidney Transplantation
6. Urinary Incontinence

12- Did you have mandatory urology classes?

1. Yes
2. No

13- Were the urology classes you attended:

1. Theorical? Yes / No
2. Practical? Yes / No
3. Case Clinical Discussion? Yes / No

14- Did you attend any Urology clerkship, beyond the required?

1. Yes
2. No

15- Have you ever considered being a Urologist, if you could choose it?

1. Yes
2. No

16- Did your teaching at Medical School influence the choice of your current specialty?

1. Yes
2. No
